# Electroacupuncture ameliorates inflammatory pain through CB2 receptor-dependent activation of the AMPK signaling pathway

**DOI:** 10.1186/s13020-024-01048-z

**Published:** 2024-12-24

**Authors:** Yuye Lan, Xianghong Jing, Ziyu Zhou, Yiqing Rao, Kaichen Wang, Renjie Qin, Yisong Wu, Jingjing Sun, Ke Zhang, Xinyue Liu, Zixiao Wang, Jiahao Xu, Minzhen zhao, Xiao Cui Yuan, Yongmin Liu, Hong Zhang, Xuefei Hu, Huilin Pan, Tengfei Hou, Man Li

**Affiliations:** 1 Department of Neurobiology, School of Basic Medicine, Tongji Medical College, Key Laboratory of Neurological Diseases of Hubei Province and National Education Ministry, Huazhong University of Science and Technology, Wuhan, 430030 China; 2 Institute of Acupuncture and Moxibustion, China Academy of Chinese Medical Sciences, Beijing, 100700 China; 3 Department of Physiology and Pathophysiology, School of Basic Medical Sciences, Xi’an Jiaotong University Health Science Center, Xi’an, 710061 China; 4 School of Clinical Medicine, Hubei University of Science and Technology, Xianning, 437000 China; 5 Institute of Acupuncture, School of Medicine, Jianghan University, Wuhan, 430056 China; 6 Department of Anesthesiology and Perioperative Medicine, The University of Texas MD Anderson Cancer Center, Houston, TX 77030 USA

## Abstract

**Background:**

Chronic inflammatory pain is a pervasive condition, and electroacupuncture (EA) is an effective treatment, but its mechanisms are not fully understood. AMP-activated protein kinase (AMPK), a key energy sensor, is involved in pain relief and EA’s effects. EA may work by increasing endocannabinoids, upregulating CB2 receptors (CB2R), and stimulating β-endorphin (β-END). This study tests if EA activates AMPK via CB2R to modulate β-END and reduce pain.

**Methods:**

The inflammatory pain model was established with Complete Freund’s adjuvant (CFA), and EA was administered daily for six consecutive days, targeting the acupoints “Zusanli” (ST36) and “Shangjuxu” (ST37). Pain sensitivity was evaluated using Von Frey filaments for mechanical thresholds and a hot plate for thermal thresholds. Ultra-high Performance Liquid Chromatography Tandem Mass Spectrometry (UPLC-MS/MS) was used to quantitatively determine the levels of endocannabinoids 2-arachidonoylglycerol (2-AG) and anandamide (AEA). The expression levels of the CB2R and β-END were measured by Western blotting, along with the activation of AMPK. Immunofluorescence double-labeling was applied to visualize AMPK activation and β-END expression within CD68-positive macrophages. The study encompassed both wild-type and CB2R gene knockout mice, elucidating the role of CB2R in EA-induced AMPK activation.

**Results:**

CFA-induced inflammatory pain model mice exhibited mechanical allodynia and thermal hyperalgesia. EA activated AMPK in the inflamed skin tissue when it exerted analgesic effect on the inflammatory pain. Pre-administration of the AMPK inhibitor Compound C significantly inhibited the effect of EA on pain relief. EA elevated β-END expression in inflamed skin tissue, which was reversed by Compound C, indicating that AMPK has a regulatory role in EA inducing β-END expression. In addition, EA significantly upregulated the levels of 2-AG, AEA and the expression of CB2Rs in the inflamed skin tissue compared with the CFA group. In wild-type mice, EA activates AMPK in macrophages, while CB2 knockout reduced EA's ability to activate AMPK in these cells.

**Conclusion:**

EA activates AMPK through CB2R, enhancing β-END expression in inflamed skin to alleviate inflammatory pain. This study reveals a new link between endocannabinoids, endorphins, and AMPK in analgesic effects of EA, highlighting the CB2R-AMPK-β-END pathway.

**Supplementary Information:**

The online version contains supplementary material available at 10.1186/s13020-024-01048-z.

## Introduction

The detrimental impact of pain on human health should not be underestimated, and it has long been a prominent focus of global healthcare. Chronic pain not only significantly disrupts individuals' daily lives but also imposes an enormous economic burden [1, 2]. Inflammatory pain, a prevalent form of chronic pain encountered in clinical practice, is commonly associated with conditions such as osteoarthritis, rheumatoid arthritis, and myofascial pain. Currently, the primary clinical management of inflammatory pain relies on non-steroidal anti-inflammatory drugs (NSAIDs) and opioid analgesics. However, NSAIDs exhibit adverse effects on the gastrointestinal tract and cardiovascular system, while opioid analgesics are associated with tolerance and addiction. Consequently, these limitations restrict applicability in clinical practice [3, 4].

Electroacupuncture (EA), a widely employed analgesic modality in clinical acupuncture, exhibits a cumulative effect when the treatment target is repeated. Randomized controlled trials have demonstrated the effectiveness of EA in relieving chronic pain associated with knee osteoarthritis, fibromyalgia pain, myofascial pain, and other inflammatory conditions [5, 6]. The combination of EA and medication demonstrates superior efficacy in managing rheumatoid arthritis pain compared to the use of Western medicine alone [7]. Adenosine monophosphate-activated protein kinase (AMPK) is a eukaryotic cellular kinase that regulates energy metabolism by modulating intracellular AMP/ATP level, thereby playing a crucial role in the modulation of various pain conditions including inflammatory pain, neuropathic pain resulting from nerve damage, postoperative pain, and chemotherapy-induced or diabetes-induced neuropathic pain [8–10]. Importantly, the pharmacological activation of AMPK not only alleviates acute pain but also effectively treats chronic pain, with its efficacy enhanced through repeated administration [11].

Studies have shown that AMPK is closely related to EA analgesia. EA can improve the symptoms of rats with acute ulcerative colitis, which may be related to promoting colonic autophagy and increasing AMPK phosphorylation level [12]. The level of AMPK gene expression in the hypothalamus of rats determines the individual differences in EA analgesia sensitivity [10]. EA may alleviate neuropathic pain caused by nerve injury by promoting AMPK/ mTOR-mediated autophagy in spinal microglia [13]. Previous study has shown that EA treatment at the “Zusanli” (ST36) and “Shangjuxu” (ST37) acupoints along the Stomach Meridian effectively alleviates Complete Freund’s Adjuvant (CFA) -induced inflammatory pain in mice [14]. It remains unclear whether repeated EA at ST36 and ST37 points can activate AMPK in peripheral skin tissue to exert analgesic effects.

EA has been shown to augment the release of endocannabinoids in CFA-induced inflamed skin tissue [15]. It also upregulates and activates the expression of CB2R on immune cells including macrophages, T lymphocytes, and keratinocytes within the skin tissue [16]. Furthermore, EA facilitates the synthesis and release of the endogenous opioid peptide β-endorphin (β-END) within these cells. The released β-END subsequently activates μ-opioid receptors on nociceptors, reducing their excitability and thereby exerting analgesic effects [17]. Literature suggests that neutrophils have the capacity to release β-END [18], yet there is currently insufficient evidence to support the notion that the release of β-END in neutrophils is modulated by CB2R. Moveover, AMPK is only found to be upstream of the CB2 receptor in macrophages, but not T lymphocytes, and keratinocytes. Within mouse peritoneal macrophages, the AMPK-mTOR-P70S6K signaling pathway is thought to play a role in the anti-inflammatory effects mediated by CB2R [19]. In addition, it is noteworthy that activation of CB2R can effectively stimulate AMPK [20–23]. The activation of AMPK can induce a phenotypic shift in macrophages from the pro-inflammatory M1 phenotype, which is associated with the release of pro-inflammatory cytokines, to the anti-inflammatory M2 phenotype, which can produce anti-inflammatory and immunosuppressive factors. This shift leads to an upregulation of endorphin expression and subsequent alleviation of inflammatory pain [24]. Therefore, CB2R-AMPK-END pathway is more likely to exist in macrophages, which should be the main target cells for EA to relieve inflammatory pain.

Based on this, the present study proposes a scientific hypothesis that EA at the ST36 and ST37 acupoints induces the release of endocannabinoids in the skin, thereby activating CB2R on immune cells such as macrophages in inflamed skin tissue to sustainably activate AMPK. Consequently, this process promotes the synthesis and release of β-END, inhibits nociceptive information transmission, and ultimately exerts a cumulative analgesic effect on inflammatory pain.

To verify the preceding hypothesis, our study commenced by examining the activation of AMPK by EA in CFA-inflamed skin tissue. Subsequently, we utilized a mouse model treated with the AMPK inhibitor Compound C to confirm the critical role of AMPK in mediating the analgesic effects of EA and its influence on β-END expression. Furthermore, we explored the upstream mechanisms by which EA activates peripheral AMPK and its analgesic effects, focusing on the influence of EA on endocannabinoids and CB2R expression in inflamed skin. Finally, we employed CB2R knockout (CB2R KO) mice to establish that EA triggers AMPK phosphorylation via CB2R activation, thereby investigating the potential of EA to activate the CB2R-AMPK-β-END pathway and produce analgesic effects.

## Materials and methods

### Animal models

Male C57BL/6 mice (8 weeks old) were purchased from Beijing Vital River Laboratory Animal Technology Co., Ltd. CB2R KO mice were purchased from Jackson Laboratory in the United States (Jackson Laboratory, JAX005786). The mice were housed in a controlled environment with a relative humidity of 60 ± 10%, temperature maintained at 22 ± 2 ℃, and subjected to a 12-h light/dark cycle. Prior approval for the animal study protocol was obtained from the Ethics Committee for Experimental Animals at Tongji Medical College, Huazhong University of Science and Technology (2022 Ethics No.3603). Before commencing the experiment, mice were acclimated in the animal facility for one week, with each cage housing 4–6 mice.

Subcutaneous injection of CFA is a widely used model for inflammatory pain. Inflammation was induced by injecting 25 μL of CFA (Sigma, St. Louis, MO, USA) subcutaneously into the dorsal surface of the left hind paw of mice using a 25-gauge hypodermic needle [25]. The injections were carried out under light anesthesia by inhalation of ether. The control group received an equivalent volume of normal saline as vehicle (Veh.) instead of CFA.

### EA treatment and AMPK inhibitor administration

The acupoint positioning in mice followed the previous study [14]. ST36 was located on the lateral side of the knee joint, approximately 2 mm below the fibular head. ST37 was situated directly beneath Zusanli (ST36), at a depth of 5 mm. On the second day after establishing a CFA model (day 2), mice in the EA group were secured inside custom-made denim mouse jackets to expose and immobilize their left lower limbs. The acupoints ST36 and ST37 were stimulated using semi-inch filiform needles. These needles, measuring 0.20 mm in diameter and 13 mm in length, were sourced from Wujiang Yunlong Medical Instruments Co., Ltd., China. The needles were inserted perpendicularly into the acupoints to a depth of 2–3 mm. The needle handle was connected to a Han's acupoint and nerve stimulator device (LH202 model) set in continuous wave mode with a frequency of 2 Hz and an intensity of 1 mA. Stimulation was administered once daily for six consecutive days, each session lasting for 30 min. Mice in both control group and model group were placed inside denim jackets using similar methods but did not receive any treatment during the EA period. Mice in the sham EA group underwent shallow subcutaneous needling without connecting to Han's acupoint and nerve stimulator device. Throughout the entire treatment process, all mice remained alert without exhibiting any signs of pain or distress. AMPK inhibitor Compound C (CC, 20 mg/kg) or its vehicle was intraperitoneally injected 30 min before EA treatment. The animals were divided into four groups in both wild type (WT) and CB2R KO C57BL/6 mice: Control group, CFA group, CFA + EA group, CFA + sham EA group; The animals were divided into four groups in WT mice: Control group, CFA group, CFA + Veh. + EA Group, CFA + CC + EA Group.

### Nociceptive behavioral tests

The mechanical pain threshold in mice was assessed using the “up and down” method [26]. Prior to the baseline measurement, the mice were habituated to the testing chamber for 30 min per day for three consecutive days. Von Frey filaments (Stoelting Company, Wood Dale, Italy) were applied vertically to the mouse's paw until it bent for a maximum of 5 s. A positive response was recorded as a rapid withdrawal or claw retraction. If a positive response (represented by the symbol ‘ × ’) occurred, a finer adjacent filament was selected for further measurement; Conversely, if there was no response (represented by the symbol ‘○’), a thicker adjacent filament was chosen for subsequent measurement. Each mouse's mechanical pain threshold was determined by averaging two measurements.

The hot plate method is utilized for detecting thermal pain latency, whereby mice are positioned on a 53℃ hot plate and their paw responses are observed and timed. The timer is stopped when the mice lift or shake their paws, indicating the thermal pain latency. To prevent tissue damage, the duration of the hot plate test is limited to 20 s [27]. The thermal pain latency of each mouse is determined by averaging three experiments conducted with a 5-min interval between each trial. All the nociceptive behavioral tests were measured after daily treatment.

### Histological evaluation

On the 7th day of CFA modeling, after the last EA treament, mice were subjected to anesthesia using 3% isoflurane, and the affected skin was rapidly dissected on ice. The excised skin samples were then fixed in a 4% paraformaldehyde solution, embedded in paraffin, and sectioned into 8-µm slices using a microtome. A subset of these sections was stained with hematoxylin and eosin (H&E) to assess tissue morphology. Epidermal thickness was measured in five randomly selected high-power fields (20 × magnification) per mouse using ImageJ software[28]. Each section was independently evaluated by two investigators in a double-blinded manner.

### Western blotting (WB)

After the last EA treament, the mice were deeply anesthetized with 1% sodium pentobarbital, and the skin of the left dorsal foot was removed and placed on ice. A strong RIPA lysate (10 μl/mg, Beyotime Biotechnology), a protease inhibitor and phosphatase inhibitor (1 mM, Wuhan Guge Biotechnology) were added to the tissue, which was then fully ground and cracked on ice for 30 min. Centrifuge the sample at 4 ℃, 12,000 rpm for 15 min and remove the supernatant after centrifugation. Protein concentration was determined using a BCA protein assay kit (Beyotime Biotechnology). Aliquots of protein samples (20 μg) were run on 10% SDS-PAGE gel and then transferred to PVDF membranes. The membrane was then blocked with 5% skimmed milk in TBST for 1 h at room temperature. The membranes were incubated with the primary antibody against p-AMPK (Thr172) (1:1000, Cell Signaling Technology), AMPK (1:1000, Cell Signaling Technology), CB2R (1:1000, Abcam), GAPDH (1:20,000, Proteintech) overnight at 4 ℃. After three washes with TBST, the membranes were incubated with secondary antibodies of goat anti-rabbit HRP or goat anti-mouse HRP (1:20,000, Wuhan Kerui Technology) for 1 h at room temperature. The protein signals were developed using Pierce ECL WB Substrate (Thermo Fisher Scientific, USA). Density analysis was performed using the ImageJ 6.0 software (National Institutes of Health, Bethesda, MD, USA).

### Immunofluorescence

After the last EA treament, mice were anesthetized with 1% sodium pentobarbital at 50 mg/kg, followed by thoracic exposure and perfusion with 37 ℃ normal saline and 4% paraformaldehyde dissolved in 0.01 M phosphate-buffer solution (PBS, pH 7.4, 4 ℃). Left dorsal foot skin tissues were harvested and postfixed at 4 ℃ for 8 h. Then, skin tissues were cryoprotected in 30% sucrose overnight, and sectioned at 15 μm.

Double immunolabeling was performed with rabbit anti-p-AMPK (Thr172, 1:250, Abcam) or rabbit anti-β-END (1:200, Abcam) and mouse monoclonal anti-CD68 antibody (1:200, Abcam) respectively. All sections were blocked for 30 min with 5% donkey serum and 0.2% Tween-20 in PBS, followed by incubation at 37℃ for 1 h then at 4℃ overnight with the primary antibody diluted in PBS containing 5% bovine serum albumin. The sections were then washed 4 times with 0.05% Tween-20 in PBS for 5 min, and incubated with a mixture of secondary antibodies: donkey anti-mouse IgG conjugated with Dynight 488 (1:600, Jackson ImmunoResearch) and donkey anti-rabbit IgG conjugated with Dynight 594 (1:600, Jackson ImmunoResearch). Sections were washed 4 times with 0.05% Tween-20 in PBS for 5 min, and then treated with the fluorescence-mounting medium to inhibit the quenching of fluorescence before being coverslipped. To ensure the validity of the results, negative controls were included by omitting the primary antibodies and with primary antibodies preabsorbed with their specific blocking peptides in the above procedures, which resulted in no positive labeling in the skin tissues.

An Olympus BX51 fluorescence microscope was used to view the sections, and images were obtained using a Qimaging Camera and QCapture software. A total of 5–6 sections from the skin were randomly selected in each mouse. The percentage of the percentage of positive area of p-AMPK or β-END/macrophages (CD68 +) was quantified by ImageJ software.

### Polymerase chain reaction (PCR) on genomic DNA

After the last EA treament, DNA was precipitated from the tail of the mouse after overnight incubation in lysis-buffer (100 mM Tric-Cl, 5 mM EDTA, 0.2% SDS, 200 mM NaCl and 0.1 mg/ml Proteinase K). The DNA pellet was resuspended in 100 μl of sterilized water and 2 μl was used for each 20 μl PCR reaction. The CB2R primers used are (GGGGATCGATCCGTCCTGTAAGTCT), (GACTAGAGCTTTGTAGGTAGGCGGG) and (GGAGTTCAACCCCATGAAGGAGTAC). The reaction components for PCR assays were as follows: 20 μl PCR Mix, 6.5 μl dd H2O, 10 μl 2 × Phanta Max Master Mix, 2 μl DNA, 0.5 μl primer 1, 0.5 μl primer 2 and 0.5 μl primer 3. It was amplified in Eppendorf Master cycler PCR instrument (Eppendorf, Hamburg, Germany) for predegeneration at 94℃ for 3 min, and then 35 cycles at 94℃ for 30 s, 60℃ for 30 s, and 72℃ 1 min, followed by a final extension at 72℃ for 5 min. The PCR products were sequenced by Sangon Biotech (Shanghai, China), after being analyzed by 1.5% agarose gel.

### The contents of 2-AG and AEA in the samples were determined by HPLC

Analysis of 2-AG and AEA was conducted using Ultra-high Performance Liquid Chromatography Tandem Mass Spectrometry (UPLC-MS/MS).The skin tissues were removed after the last EA treament. The skin tissues were weighed, ten times methanol was added and swirled for 30 s, followed by ultrasonic treatment for 20 min. After centrifugation at 13,000 rpm for 10 min, the supernatant was removed and filtered with 0.22 μm filter membrane, followed by quantification with UPLC-MS/MS. Analyses were conducted on UPLC (I-Class)-MS (XEXO TQ-MS) and MassLynx V4.1 workstation. The mass spectrum detection parameters of the components to be measured were: Source Voltages: 3.00 kV; Source Temperature: 450 ℃; Gas Flow: 800 L/Hr; Cone: 50 L/Hr. Analytical LC separations were performed on Waters ACQUITY UPLC BEH-C18 (2.1 × 50 mm, 1.7 μm) with a flow rate of 0.3 mL/min and a column temperature of 35 ℃ using a gradient of acetonitrile (eluent B) and water (eluent A) both containing 0.1% formic acid. The gradient was as follows: 2% eluent B for 1.0 min; 2–100% B from 1.0 to 2.0 min and held at 100% from 2.0 to 3.5 min. From 3.5 to 4.0 min, the column was re-equilibrated to 2% B and conditioned from 4.0 to 6.0 min at 2% B.

### Identification of gene knockout (CB2R KO) mice

PCR electrophoresis and WB test were used to identify whether CB2R gene was knocked out in mice. As shown in Supplementary Fig. 1, the electrophoretic diagram of PCR amplification products of CB2R is on the left, and the DNA Marker indicator diagram is on the right. The 385 bp band represented wild-type mice (WT) and the 550 bp band represented CB2R KO mice, and both bands were identified as heterozygotes. According to the electrophoretic map of PCR amplification products of CB2R gene in supplementary Fig. 1, lane M was the mouse tail DNA Marker, lane WT was the positive control, lane H2O was the negative control, and lane A1-A8 was the sample. Therefore, lanes A1-3, A5 and A8 were homozygous mice, that is, CB2R KO mice; Lane A4, A6 and A7 were heterozygous mice; As shown in Supplementary Fig. 2, WB experiment was performed on the dorsal hindpaw skin of wild-type and CB2R KO mice. The experimental results showed that compared with wild-type mice, the expression level of CB2R in the dorsal hindpaw skin of CB2R KO mice was significantly reduced (Supplementary Fig. 2, P < 0.01).

### Statistical analysis

The experimental results were expressed as mean ± SEM. Two-way ANOVA was used to analyze the mechanical pain threshold and thermal pain latency. One-way ANOVA and Newman-Keuls post hoc test (SPSS, version 11.0) or T-test were used to analyze other data. P < 0.05 was considered statistically significant.

## Result

### EA exerts analgesic effects by activating AMPK in the inflammatory skin tissue

**Fig. 1:**
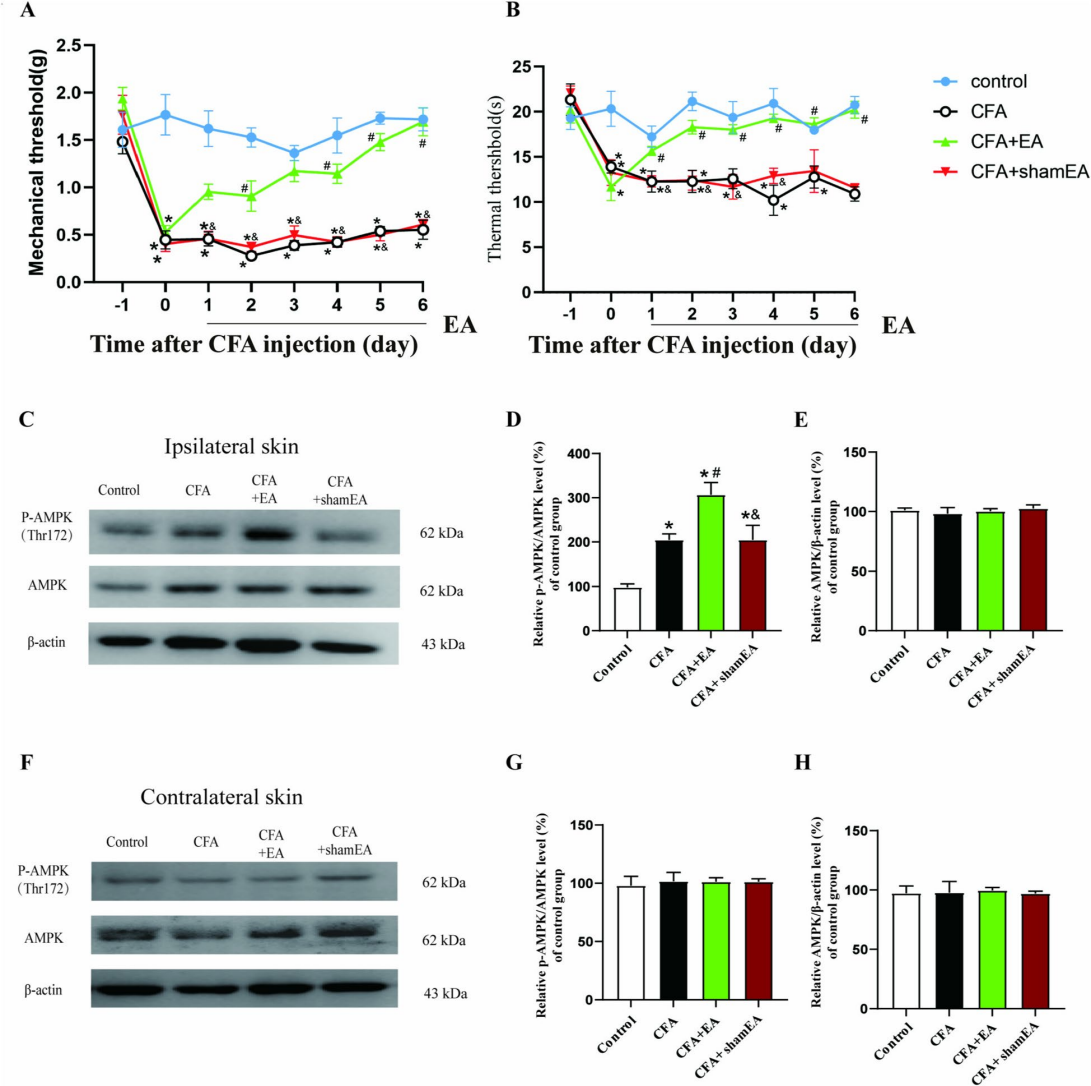
Effect of EA on pain behavior and AMPK activation in inflamed skin tissue. **A** Mechanical pain threshold. **B** thermal pain latency. The data was shown as mean ± SEM (n = 10). Two-way ANOVA was used to analyze the data. **C** AMPK phosphorylation level in affected (ipsilateral) dorsal hindpaw inflamed skin tissue. **D** A statistical histogram of p-AMPK percentage in total AMPK.**E** statistical histogram of total AMPK percentage in β-actin. **F** AMPK phosphorylation level in normal skin tissue of the healthy side (contralateral) dorsal hindpaw.**G** A statistical histogram of p-AMPK percentage to total AMPK. **H** A statistical histogram of total AMPK percentage to β-actin. The data is shown as mean ± SEM (n = 4).One-way ANOVA was used to analyze the data. *P < 0.05 compared with Control group; ^#^P < 0.05 compared with CFA group; ^&^P < 0.05 compared with CFA + EA group

After daily treatment, the pain behavior test was used for assessing EA efficacy. Results showed that on the first day after modeling, compared with the Control group, the mechanical and thermal pain latency of the affected hind paw of mice in CFA, CFA + EA and CFA + sham EA groups were significantly lower than those in the Control group (Fig. 1A, B, P < 0.05), which proved that the CFA modeling was successful and comparable. Compared with the Control group, mice in the CFA group had significantly lower mechanical pain threshold (Fig. 1A, P < 0.05) and shorter thermal pain latency (Fig. 1B, P < 0.05); In comparison with the CFA group, mice in the EA group but not in the sham EA group demonstrated significantly elevated mechanical pain threshold (Fig. 1A, P < 0.05) and extended thermal pain latency (Fig. 1B, P < 0.05). Moreover, the pain threshold progressively increased in correlation with the duration of EA treatment (Fig. 1). These findings suggested that EA has an analgesic effect.

In order to determine whether EA affects the inflammatory response, we used H&E staining to investigate the pathological changes of the inflammed skin of the left dorsal foot. It revealed significant epidermal hyperplasia, significantly increased telangiectasia, and inflammatory cell infiltration in CFA, CFA + EA and CFA + sham EA groups (Supplementary Fig. 3 A). EA significantly improved the histologic changes caused by CFA. The skin thickness of the CFA group was significantly increased compared with the control group, which was decreased in the CFA + EA group ((Supplementary Fig. 3B, P < 0.01).

The phosphorylation level of AMPK was assessed on day 7 following the completion of EA. WB analysis of the ipsilateral dorsal hind paw, which was the affected side, revealed significant differences in the ratio of phosphorylated AMPK (p-AMPK) to total AMPK. Specifically, the p-AMPK at the α-subunit Thr172 site, normalized to total AMPK, exhibited a marked increase in the CFA group, CFA + EA group and CFA + sham EA group when compared to the control group (Fig. 1C, D, P < 0.05). However, the analysis did not reveal any significant alterations in the overall levels of AMPK proteins (Fig. 1E, P > 0.05). It indicated that CFA activated AMPK in inflamed skin tissue. Compared with the CFA group, EA but not sham EA significantly increased p-AMPK /AMPK percentage of EA group (Fig. 1C, D, P < 0.05). Compared with CFA + EA group, AMPK activation was lower in CFA + sham EA group (Fig. 1C, D, P < 0.05). Importantly, there was no significant difference in the expression level of p-AMPK in normal skin of the healthy (contralateral) side dorsal hindpaw among all groups (Fig. 1F–H, P > 0.05), indicating that EA specifically activated AMPK in inflamed skin tissue.

**Fig. 2:**
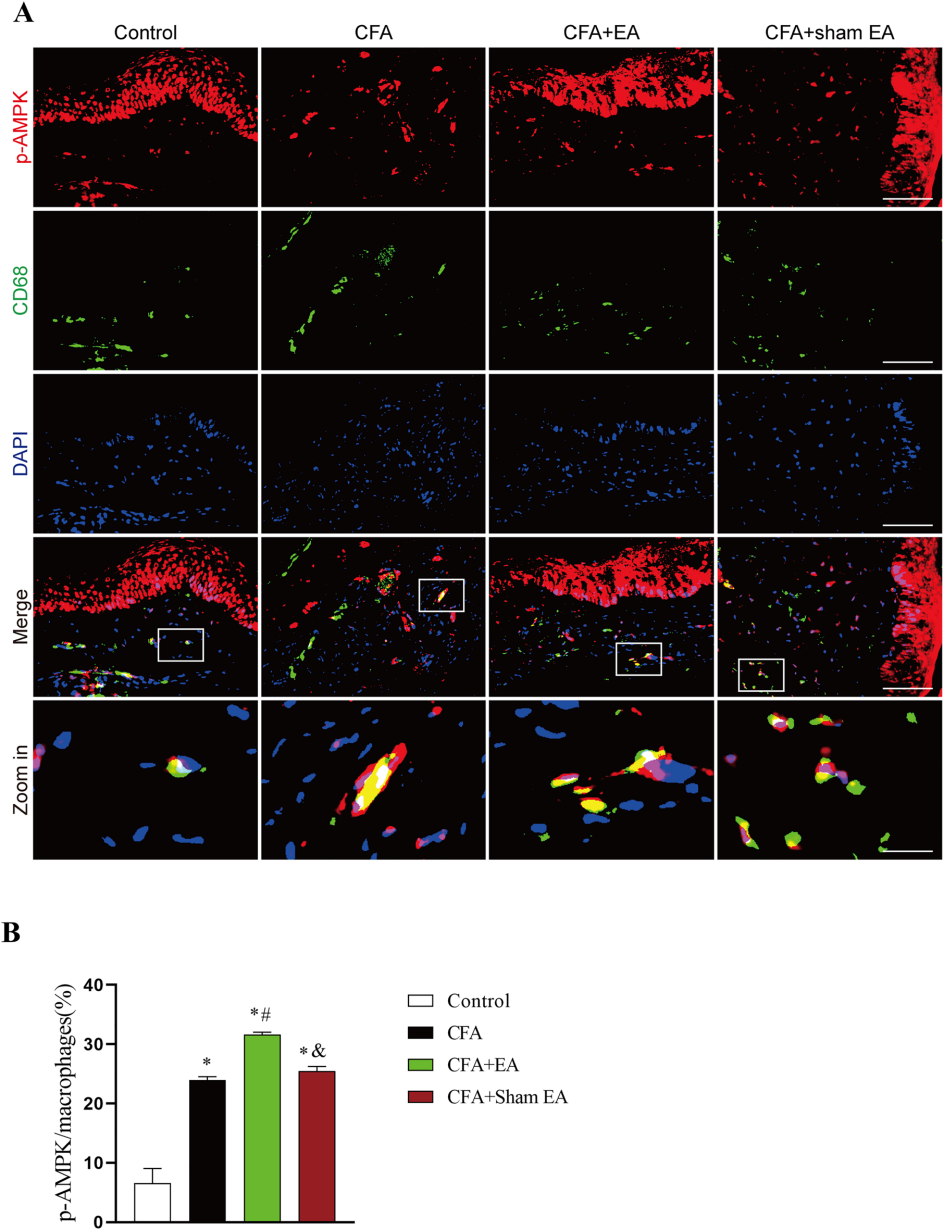
Effect of EA on AMPK activation in macrophages in inflamed skin tissue. **A** Represents the double-labeled fluorescence results of macrophages (CD68 +) and p-AMPK in inflamed skin tissue. Macrophages (CD68 +) and p-AMPK had double labeling. The scale bars are 50 μm and 10 μm (Zoom in). **B** A statistical histogram of the percentage of positive area of double-labeled cells/macrophages. The data is shown as mean ± SEM (n = 4). One-way ANOVA was used to analyze the data. *P < 0.05 compared with Control group; ^#^P < 0.05 compared with the CFA group; ^&^P < 0.05 compared with the CFA + EA group

As a kind of non-specific immune defense cells, macrophages are widely found in blood vessel walls and loose connective tissues, and play a key role in CFA-induced inflammatory pain[29].To explore AMPK activation in macrophages during EA treatment of inflammatory pain, p-AMPK expression was observed by immunofluorescence double-labeling technique. Macrophages of mice in each group were mainly distributed in the dermis, and the cytoplasm showed light green fluorescence (Fig. 2A). p-AMPK positive cells were mainly distributed in all skin layers, and their nuclei and cytoplasm showed red fluorescence (Fig. 2A), while macrophages and p-AMPK double-labeled positive cells showed yellow fluorescence (Fig. 2A).

Compared with the Control group, the percentage of positive area of macrophages expressing p-AMPK in the inflamed skin tissue of the CFA group, CFA + EA group and CFA + sham EA group was significantly upregulated (Fig. 2B, P < 0.05). The results showed that CFA-induced inflammatory pain significantly increased the expression of p-AMPK in macrophages that had infiltrated the inflamed skin tissue. This increase in p-AMPK expression corresponds to an enhanced activation of the AMPK pathway. In addition, compared with the CFA group, the expression of p-AMPK in macrophages of inflamed skin tissue in CFA + EA group was significantly upregulated (Fig. 2B, P < 0.05), indicating that EA could further promote AMPK activation in macrophages (Fig. 2B, P < 0.05). There was no significant difference in the percentage of positive area of double-labeled cells in the CFA + sham EA group compared with the CFA group (P > 0.05), but it was significantly lower than that in the CFA + EA group, suggesting that sham EA could not further promote AMPK activation in macrophage. The data indicate that inflammatory pain induced by CFA is associated with an increased activation of AMPK in macrophages that have infiltrated the inflamed skin. Furthermore, our findings suggested that EA may augment AMPK activation in macrophages of the affected side's inflamed skin, potentially contributing to the analgesic effects observed in our study.

**Fig. 3:**
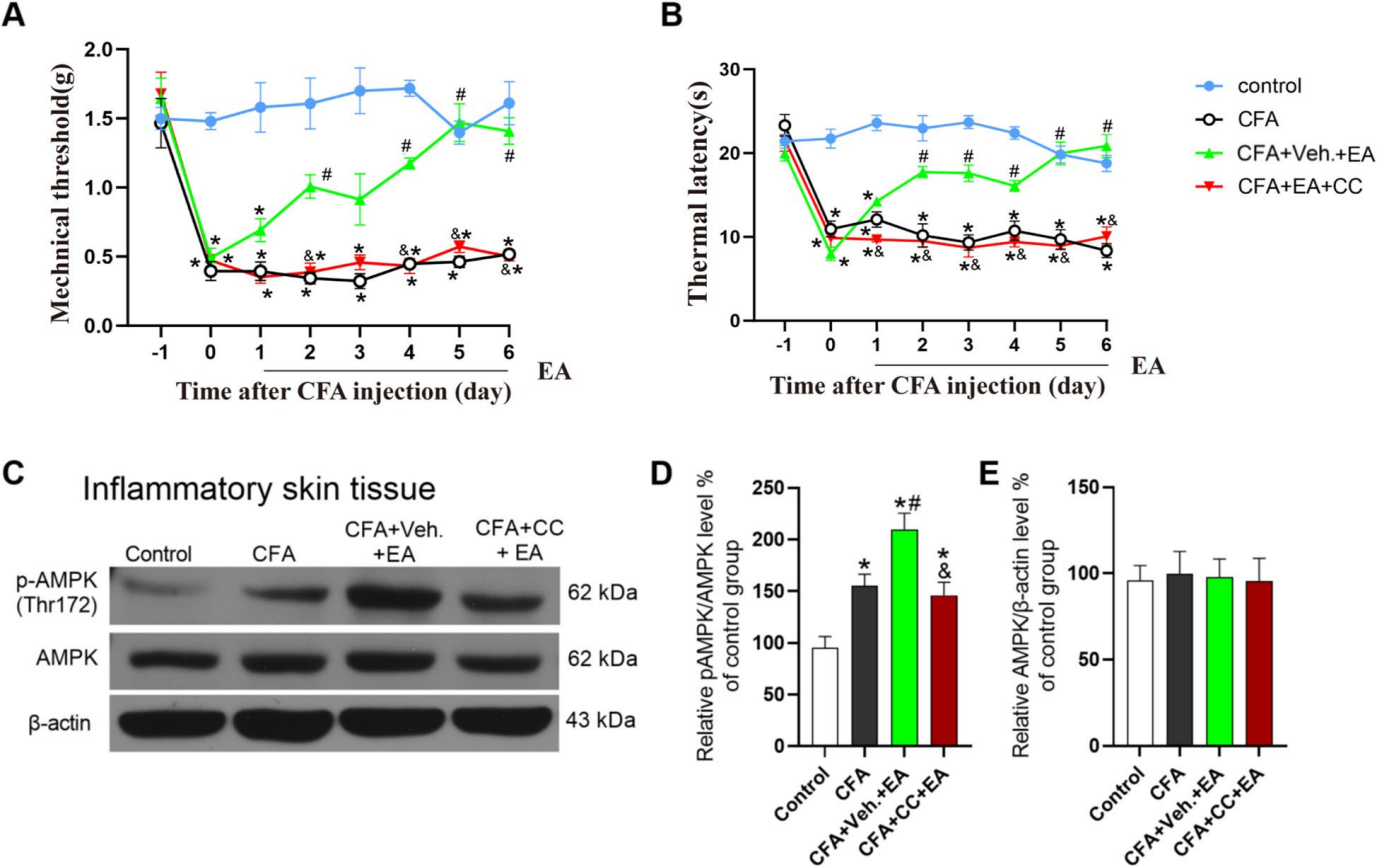
AMPK inhibitor Compound C reversed the analgesic effect of EA and the EA-mediated AMPK activation in inflamed skin tissue. **A** Mechanical pain threshold. **B** thermal pain latency. The data was shown as mean ± SEM (n = 8). Two-way ANOVA was used to analyze the data. *P < 0.05 compared with Control group; ^#^P < 0.05 compared with CFA group; ^&^P < 0.05 compared with CFA + EA group. **C** The gel representation of p-AMPK and AMPK in WB. **D** A statistical histogram of the percentage of p-AMPK in total AMPK. **E** A statistical histogram of the percentage of total AMPK in β-actin. The data is shown as mean ± SEM (n = 4). One-way ANOVA was used to analyze the data. *P < 0.05 compared with Control group; ^#^P < 0.05 compared with CFA group; ^&^P < 0.05 compared with CFA + Veh. + EA group

On the 2–7 days after CFA modeling, mice were intraperitoneally injected with AMPK inhibitor Compound C 30 min before EA treatment assess the potential impact of AMPK inhibitor on the analgesic effects of EA. In comparison to the CFA group, the CFA + Veh. + EA group demonstrated a significant elevation in both mechanical and thermal pain latency (Fig. 3A, B, P < 0.05). Conversely, the CFA + CC + EA group exhibited no significant alterations in either mechanical or thermal pain latency (Fig. 3A, B, P > 0.05). Furthermore, when contrasted with the CFA + Veh. + EA group, there was a marked reduction in both mechanical and thermal pain latency in CFA + CC + EA group (Fig. 3A, B, P < 0.05). These results suggested that the intraperitoneal administration of Compound C attenuated the analgesic effects of EA on mechanical allodynia and thermal hyperalgesia induced by CFA.

On the 7th day post-modeling, the affected skin tissue was analyzed using WB. The findings revealed that, in comparison to the Control group, the percentage of p-AMPK /AMPK was significantly elevated in the CFA, CFA + Veh. + EA, and CFA + CC + EA groups (Fig. 3C, D, P < 0.05). However, there was no significant change in the total AMPK protein levels (Fig. 3E, P > 0.05). When compared to the CFA group, the percentage of p-AMPK/AMPK in the CFA + Veh. + EA group was significantly higher (Fig. 3C, D, P < 0.05), whereas the percentage in the CFA + CC + EA group remained unchanged (Fig. 3C, D, P > 0.05). Notably, the p-AMPK /AMPK percentage in the CFA + CC + EA group was significantly reduced compared to the CFA + Veh. + EA group, indicating that the intraperitoneal administration of Compound C counteracted the activation of AMPK in the inflamed skin tissue.

### EA promotes the expression of β-END in inflamed skin tissue by activating AMPK

**Fig. 4:**
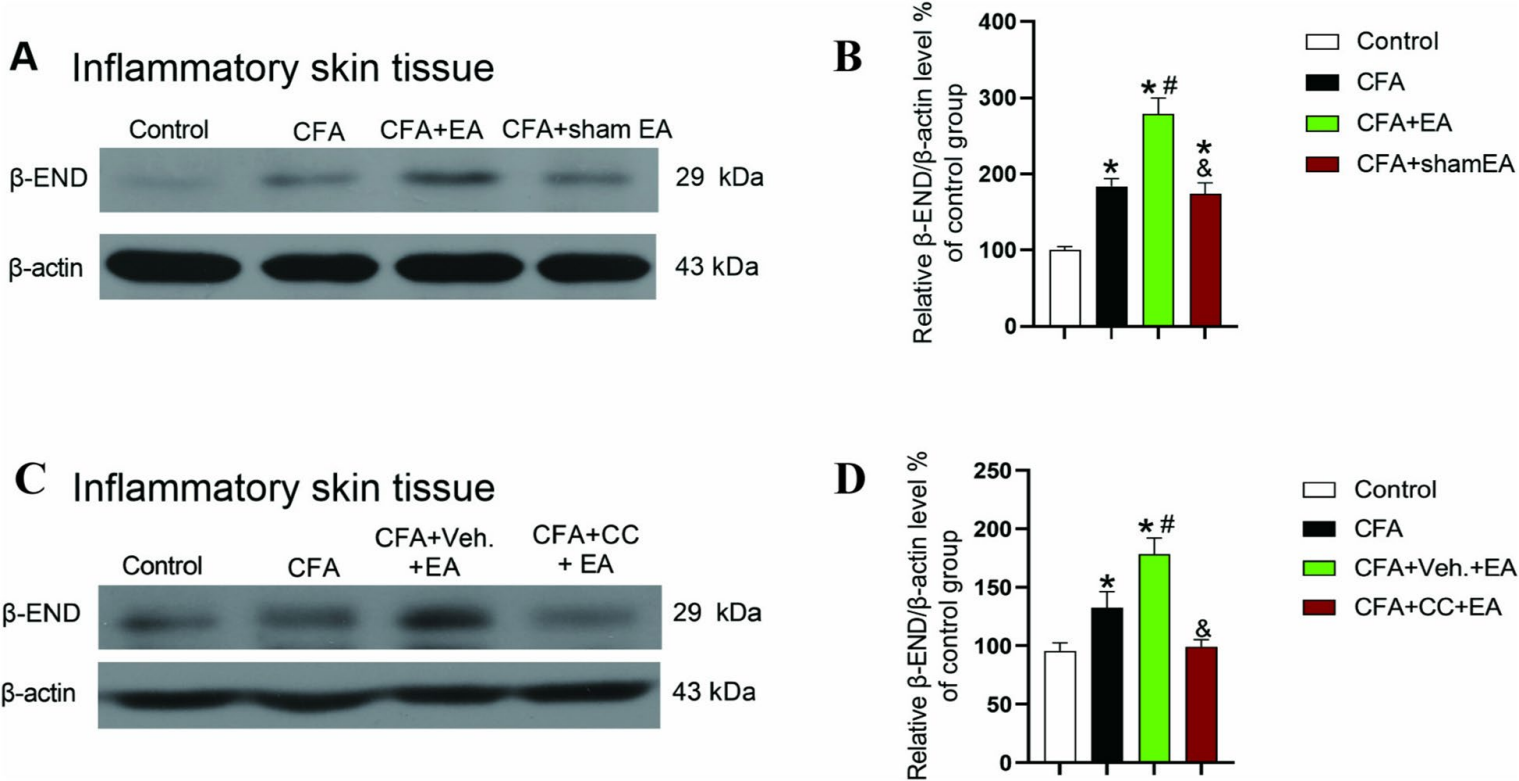
The effect of AMPK inhibitor Compound C reversed the effect of EA on promoting β-END expression in inflamed skin tissue. **A** Gel representation of β-END expression levels in skin tissue in Control, CFA, CFA + EA and CFA + sham EA group. **B** A statistical histogram of β-END expression levels in skin tissue. The data is shown as mean ± SEM (n = 4). **C** Gel representation of β-END expression levels in inflamed skin tissue of Control, CFA, CFA + Veh. + EA and CFA + CC + EA group. **D** A statistical histogram of β-END expression level in skin tissue. The data is shown as mean ± SEM (n = 4). One-way ANOVA was used for all analysis. *P < 0.05 compared with Control group; ^#^P < 0.05 compared with CFA group; ^&^P < 0.05 compared with the CFA + EA or CFA + Veh. + EA group

To determine whether EA promotes the expression of β-END in the inflamed skin tissue, WB test was used to detect its protein level. The results showed that, compared with the Control group, β-END expression in the CFA group, CFA + EA group and CFA + sham EA group was significantly increased (Fig. 4A, B, P < 0.05). Compared with the CFA group, the β-END expression in the CFA + EA group was significantly increased (Fig. 4A, B, P < 0.05). Compared with the CFA group, there was no significant change in the CFA + sham EA group (Fig. 4A, B, P > 0.05). The expression of β-END in the CFA + sham EA group was significantly lower than that in CFA + EA group (Fig. 4A, B, P < 0.05). The above experimental results showed that the inflammatory response leads to an increase in tissue β-END expression, and EA promotes this response.

**Fig. 5:**
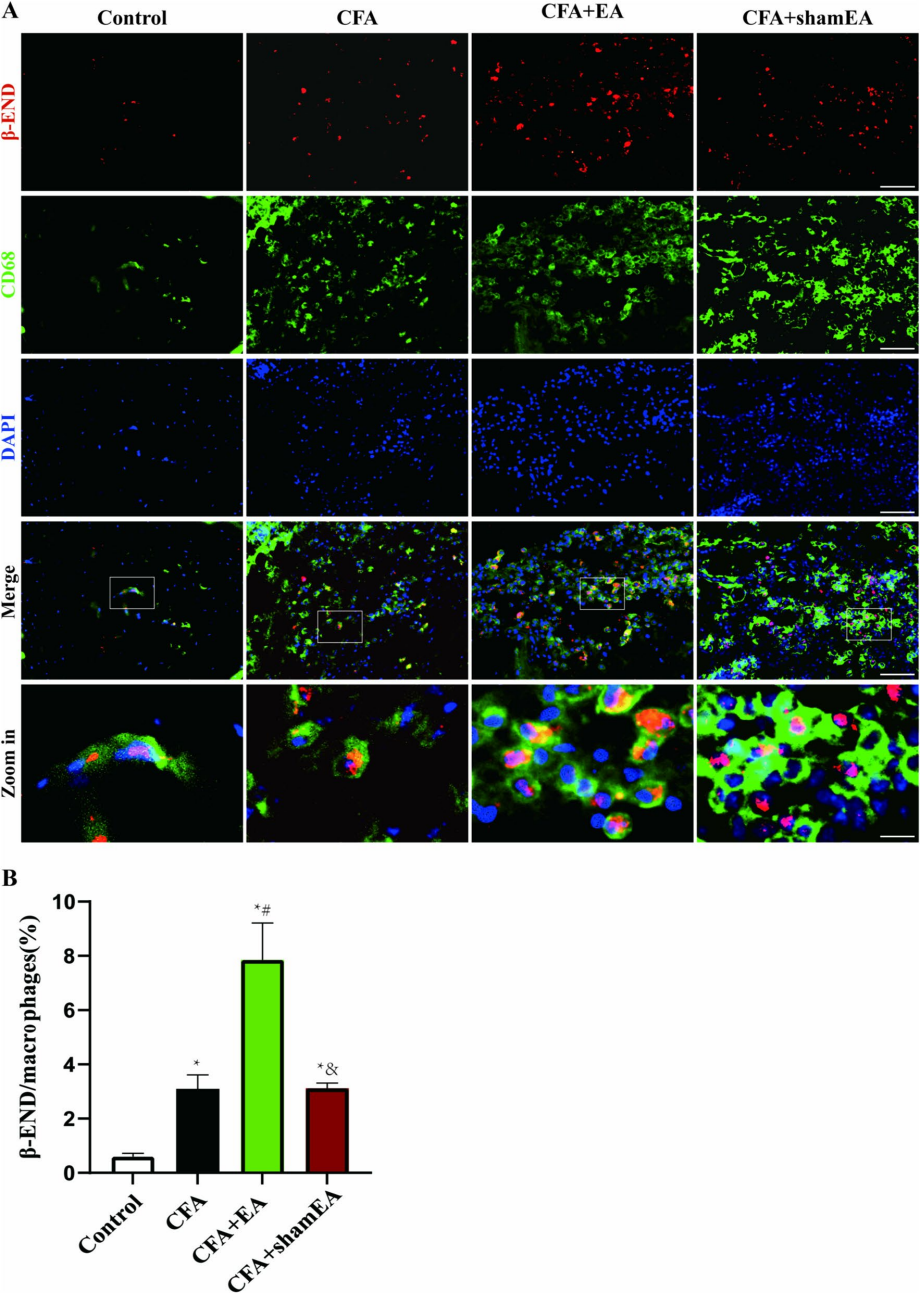
Effects of EA on macrophages and β-END expression in the inflamed skin tissue. **A** Double labeling of β-END and macrophage (CD68 +) in skin tissue with scale bars of 50 µm and 10 µm (Zoom in). **B** A statistical histogram of the percentage of positive area of macrophages (CD68 +) and β-END double-labeled cells/macrophages (CD68 single label) in the skin tissue of WT mice. The data is shown as mean ± SEM (n = 4). One-way ANOVA was used for analysis. *P < 0.05 compared with Control group; ^#^P < 0.05 compared with the CFA group; ^&^P < 0.05 compared with the CFA + EA group

To further clarify that EA enhanced expression of the analgesic substance β-END in the macrophages of inflamed skin tissue, we tested the percentage of the β-END expressing macrophages. The results showed that macrophages (CD68 +) and β-END positive cells were mainly distributed in the dermis, and the cytoplasm of macrophages showed light green fluorescence, the cytoplasm of β-END positive cells showed red fluorescence, and the double-labeled positive cells showed yellow fluorescence (Fig. 5A). Compared with the Control group, the percentage of the β-END expressing macrophages in CFA group was significantly upregulated (Fig. 5B, P < 0.05). Compared with CFA group, the percentage of the β-END expressing macrophages in CFA + EA group was significantly upregulated (Fig. 5B, P < 0.05). Compared with the CFA group, the percentage of the β-END expressing macrophages in the CFA + sham EA group had no significant change (Fig. 5B, P > 0.05). The percentage of the β-END expressing macrophages in CFA + sham EA group was significantly lower than that in the CFA + EA group (Fig. 5B, P < 0.05). The above results suggested that EA can up-regulate the expression of β-END in macrophages infiltrated in inflamed skin tissue.

To confirm whether EA promotes the expression of β-END by activating AMPK in the inflamed skin tissue, WB experiment was used to investigate whether AMPK inhibitor Compound C could reverse the effect of EA on the expression of β-END. Compared with the Control group, the expression level of β-END in the CFA group and CFA + Veh. + EA group was significantly increased (Fig. 4C, D, P < 0.05). Compared with the CFA group, the expression level of β-END in the CFA + Veh. + EA group was significantly increased (Fig. 4C, D, P < 0.05). Compared with the CFA + Veh. + EA group, the expression level of β-END in CFA + CC + EA group was significantly decreased (Fig. 4C, D, P < 0.05). There was no significant difference in β-END expression level between CFA + CC + EA and CFA group (Fig. 4 C, D, P > 0.05). These results indicated that inhibiting AMPK reversed the EA induced up-regulation of β-END expression in inflamed skin tissue. It is suggested that EA may promote the expression of β-END in inflamed skin tissue by activating AMPK, thus exerting analgesic effect, which may be the downstream of EA mediated AMPK activation.

### EA significantly upregulated the content of endocannabinoids and the expression of CB2 receptor in inflammatory skin tissue

**Fig. 6:**
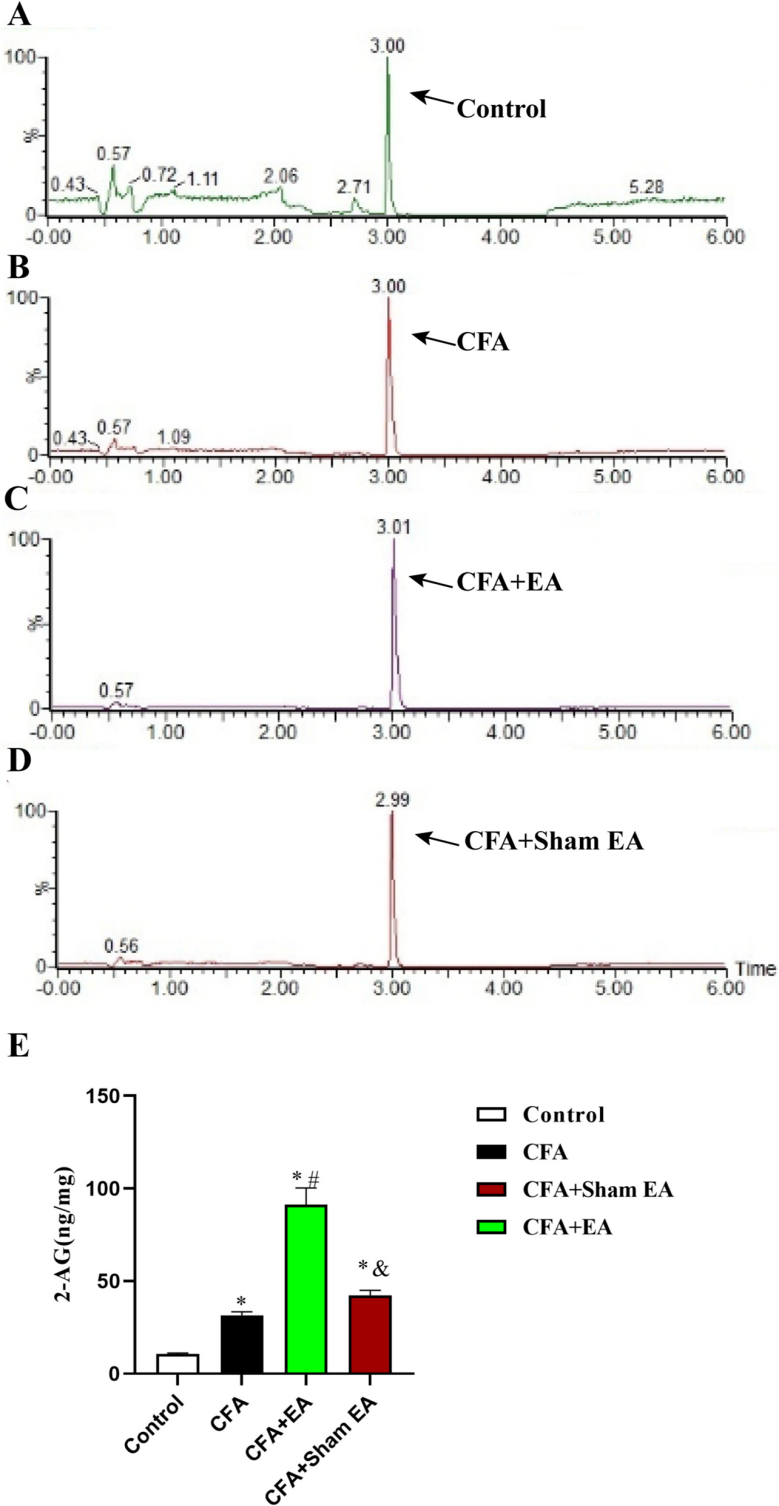
Effect of EA on 2-AG levels in the inflammatory skin tissue of the affected side of the dorsal hindpaw in mice. **A**–**D** The chromatogram of 2-AG in the skin tissue of affected side with dorsal hindpaw inflammation in the Control group, CFA group, CFA + EA group, and CFA + sham EA group. The arrow indicates the 2-AG peak. **E** A statistical histogram of the 2-AG levels. Data were expressed as mean ± SEM (n = 8). One-way ANOVA was used to analyze the data. *P < 0.05 compared with the Control group; ^#^P < 0.05 compared with CFA group; ^&^P < 0.05 compared with the CFA + EA group

**Fig. 7:**
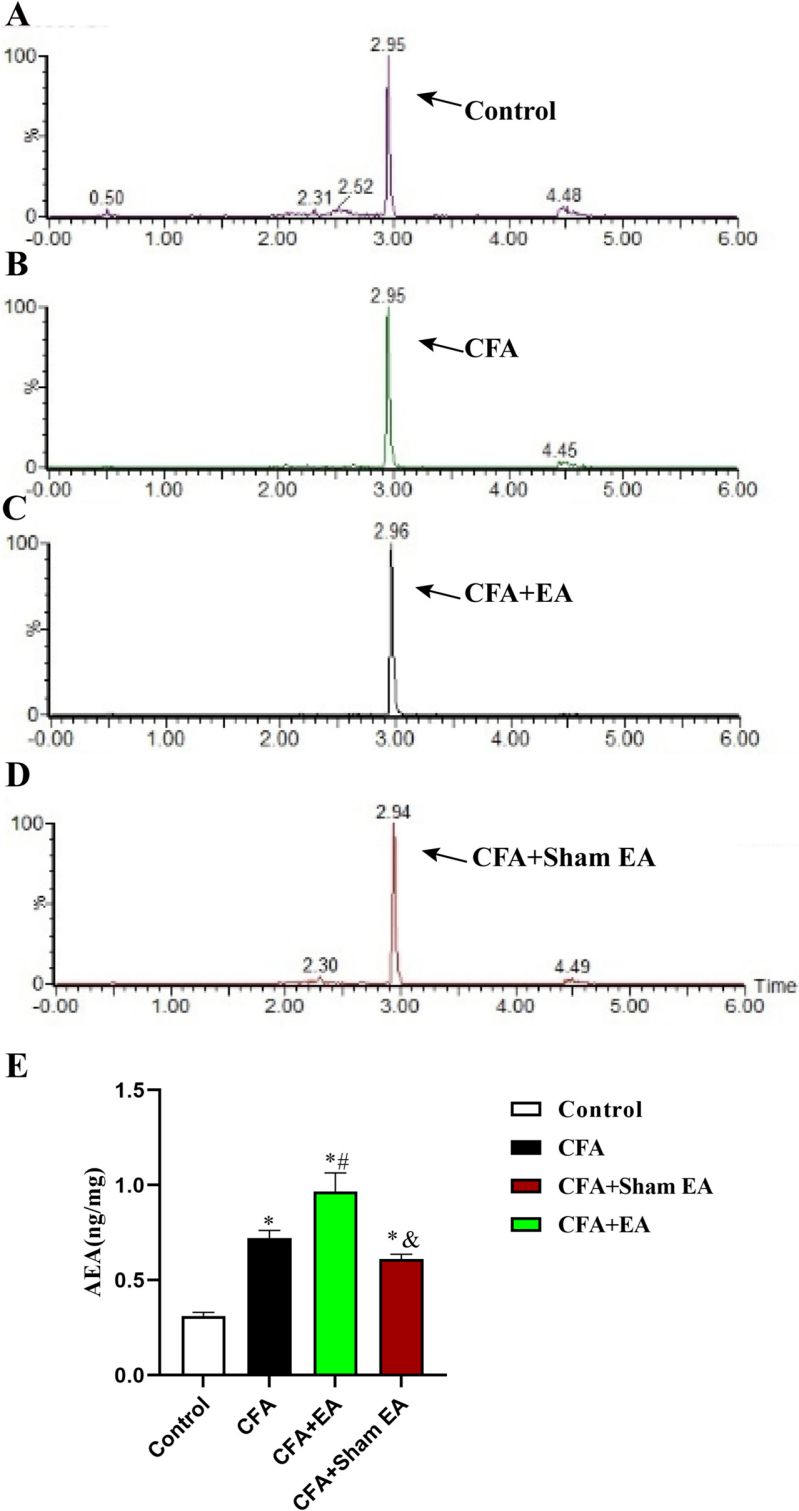
Effect of EA on AEA levels in the inflammatory skin tissue of the affected side of the dorsal hindpaw in mice. **A**–**D** The chromatogram of AEA in the skin tissue of affected side with dorsal hindpaw inflammation in the Control group, CFA group, CFA + EA group, and CFA + sham EA group. The arrow indicates the AEA peak. **E** A statistical histogram of the AEA levels. The data is shown as mean ± SEM (n = 8). One-way ANOVA was used to analyze the data. *P < 0.05 compared with the Control group; ^#^P < 0.05 compared with the CFA group; ^&^P < 0.05 compared with CFA + EA group

Chromatography was used to analyze the endocannabinoids 2-AG and AEA in skin tissue (Figs. 6, 7, P < 0.05). The results showed that compared with the Control group, the levels of 2-AG and AEA in the inflamed skin tissue of the CFA group, CFA + EA group and CFA + sham EA group were all upregulated (Figs. 6E, 7E, P < 0.05), indicating that the CFA can upregulate the contents of endocannabinoids in the inflamed skin tissue. Compared with the CFA group, the levels of 2-AG and AEA in the inflamed skin tissue of the CFA + EA group were upregulated (Figs. 6E, 7E, P < 0.05), indicating that EA further upregulated the levels of endocannabinoid in the inflamed skin tissue. Compared with the CFA group, the contents of 2-AG and AEA in inflamed skin tissue of the CFA + sham EA group had no significant change (Figs. 6E, 7E, P > 0.05). Compared with the CFA + EA group, the CFA + sham EA group had significantly lower levels of 2-AG and AEA in inflamed skin tissue (Figs. 6E, 7E, P < 0.05), indicating that sham EA could not further up-regulate the contents of endocannabinoids in inflamed skin tissue.

**Fig. 8:**
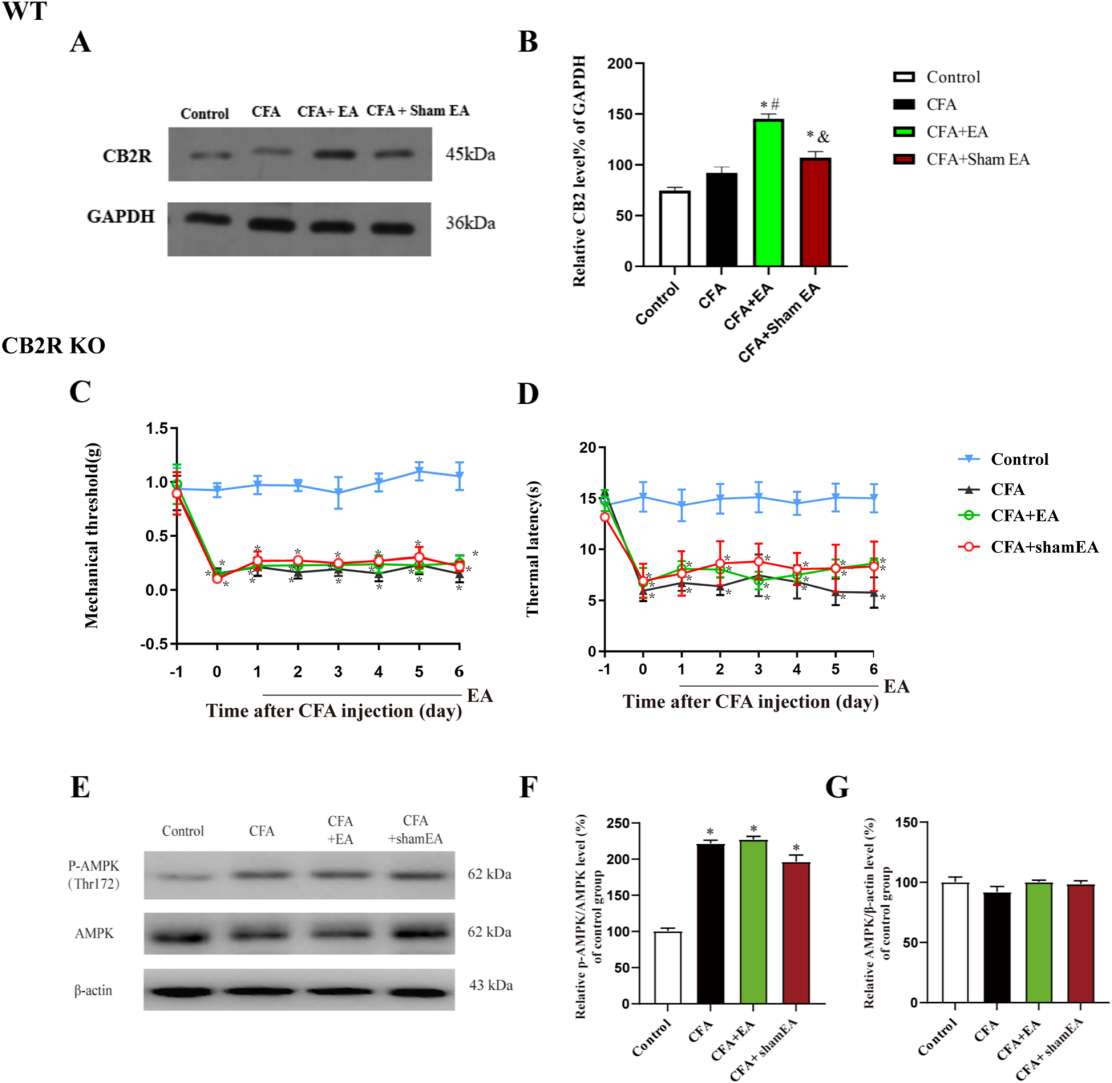
Effect of EA on the expression of CB2R and CB2R KO reversed the analgesic effect of EA and inhibited EA induced AMPK activation in inflamed skin tissue. **A** A gel representation of the effect of EA on the expression of CB2R**. B** Statistical histograms of relative quantification of CB2R protein of Wild type (WT) mice. The data is shown as mean ± SEM (n = 3). **C** The mechanical pain threshold of the affected side of CB2R KO mice. **D** The thermal pain latency of the affected side of CB2R KO mice, and the data is shown as mean ± SEM (n = 10). *P < 0.05 compared with the Control group. **E** A gel representation of the effect of EA on the level of AMPK phosphorylation in local inflamed skin tissue of CB2R KO mice. **F** A statistical histogram of the percentage of p-AMPK in total AMPK. **G** A statistical histogram of the percentage of total AMPK in β-actin. The data is shown as mean ± SEM (n = 4). One-way ANOVA was used to analyze the data of **B**, **F**, **G**. Two-way ANOVA was used to analyze the data of C, D. *P < 0.05 compared with Control group; ^#^P < 0.05 compared with CFA group; ^&^P < 0.05 compared with CFA + EA group

Concurrently, WB analysis was conducted to assess CB2R expression in the skin tissue. The WB analysis indicated variable expression of CB2R protein among the Control, CFA, CFA + EA and CFA + sham EA group (Fig. 8A, P < 0.05). The percentage of gray value of CB2R and GAPDH in the above four groups was evaluated, and it was found that there was no significant change in CB2R protein expression in the CFA group compared with the Control group (Fig. 8B, P > 0.05). However, the expression level of CB2R protein in inflamed skin tissue was significantly increased in the CFA + EA group and CFA + sham EA group (Fig. 8B, P < 0.05). Compared with the CFA group, the expression level of CB2R protein in the inflamed skin tissue in the CFA + EA group was significantly increased (Fig. 8B, P < 0.05), while there was no significant change in the CFA + sham EA group (Fig. 8B, P > 0.05). The expression of CB2R protein in the CFA + sham EA group was significantly lower than that in the CFA + EA group (Fig. 8B, P < 0.05).

These results indicate that the expression level of CB2R protein and the endocannabinoids 2-AG and AEA in the affected skin tissue can be significantly upregulated when EA exerts analgesic effect.

### CB2R KO reverses the analgesic effect of EA and inhibits EA-induced AMPK activation in inflammatory skin macrophages

To substantiate the role of the CB2R in the analgesic effects of EA and its mediation of AMPK activation, we employed a genetic approach utilizing CB2R KO mice. This methodology allows for a direct assessment of CB2R’s contribution to the observed therapeutic responses. The identification of CB2R KO mice was detailed in the supplementary materials. Pain threshold test results showed that on the first day after CFA modeling, the mechanical pain threshold and thermal pain latency of mice in CFA group, CFA + EA group and CFA + sham EA group were significantly lower than those in the Control group (Fig. 8C, D, P < 0.05), indicating that CB2R KO mice with inflammatory pain were successfully induced by CFA. Compared with the CFA group, EA no longer relieved mechanical allodynia and thermal hyperalgesia of modeling mice (Fig. 8C, D, P > 0.05), suggesting that CB2R KO significantly inhibited the analgesic effect of EA.

WB experiment results showed that compared with the Control group, the activation level of AMPK in the inflamed skin tissue in the CFA group, CFA + EA group and CFA + sham EA group was increased (Fig. 8E, F, P < 0.05), while the total AMPK protein level was not significantly changed (Fig. 8G, P > 0.05). Compared with the CFA group and CFA + sham EA group, there was no significant change in AMPK activation in the CFA + EA group (Fig. 8E, F, P > 0.05), suggesting that CB2R KO significantly inhibited the EA-mediated activation of AMPK in inflamed skin tissue.

**Fig. 9:**
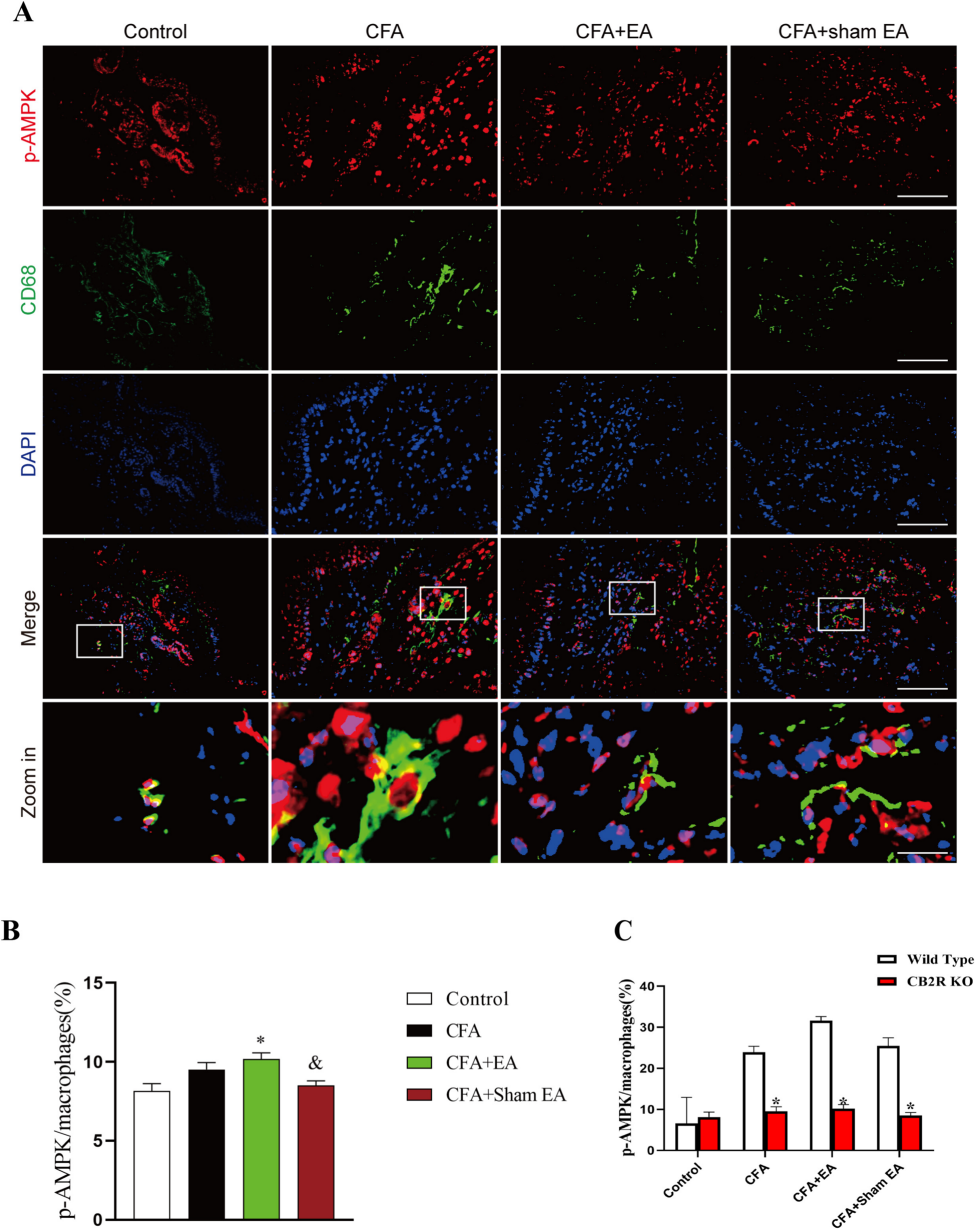
CB2R KO inhibits the activation of AMPK by EA in macrophages of inflamed skin tissue. **A** The double-labeled fluorescence results of macrophages (CD68 +) and p-AMPK in the inflamed skin tissue of CB2R KO mice. The macrophages (CD68 +) and p-AMPK had double labeling. The scale bars are 50 μm and 10 μm (Zoom in). **B** A statistical histogram of the percentage of positive area of double-labeled cells/macrophages. The data is shown as mean ± SEM (n = 4). One-way ANOVA was used to analyze the data. *P < 0.05 compared with Control group; ^#^P < 0.05 compared with the CFA group; ^&^P < 0.05 compared with CFA + EA group. **C** A statistical histogram of the percentage of positive area of double-labeled cells/macrophages between WT and CB2R KO groups. The data is shown as mean ± SEM (n = 4). T-test analysis was used to analyze the data. *P < 0.05 compared with WT mice

Furthermore, we investigated whether CB2R participated in EA-mediated activation of AMPK in macrophages infiltrated in inflamed skin tissue (Fig. 9A). After systemic knockout of CB2R, the expression of p-AMPK in macrophages of inflamed skin tissue in the CFA group was not significantly upregulated compared with that in Control group (Fig. 9B, P > 0.05). In addition, the percentage of p-AMPK in macrophages of the CFA + EA group was significantly increased compared with that in Control group (Fig. 9B, P < 0.05), but there was no significant difference compared with CFA group (Fig. 9B, P > 0.05). It is suggested that the systemic knockout of CB2R can inhibit EA-mediated AMPK activation in macrophages. Compared with the CFA + EA group, the percentage of double-labeled cells in the CFA + sham EA group significantly decreased (Fig. 9B, P < 0.05), suggesting that after CB2R KO, EA still had a certain effect on promoting AMPK activation in the inflamed skin tissue.

To investigate the difference in AMPK activation in macrophages of inflamed skin tissue after CB2R KO in each group of mice, we compared the percentage of p-AMPK expressing macrophages between WT (Data from Fig. 2) and CB2R KO mice. The results showed that the percentage of p-AMPK expressing macrophages in the CFA group, CFA + EA group and CFA + sham EA group with CB2R KO was significantly lower than that of WT mice (Fig. 9C, P < 0.05). There was no significant difference in the percentage of p-AMPK expressing macrophages between the WT and CB2R KO Control groups (Fig. 9C, P > 0.05). These results further suggested that systemic CB2R KO significantly inhibited the activation of AMPK by CFA and EA in macrophages of inflamed skin tissue.

## Discussion

### The significance of fundamental research on EA analgesia

Pain is a critical issue that significantly impacts human health. While opioid analgesics have demonstrated substantial efficacy in relieving pain, their repeated use can lead to undesirable consequences, such as tolerance and addiction. The opioid abuse epidemic in the United States has escalated into a severe public health crisis. In light of this, both the National Institutes of Health and the American Medical Association have endorsed non-pharmacological interventions, such as acupuncture, as effective treatments for chronic pain [30].

In the past three decades, numerous domestic and international clinical trials have consistently demonstrated the efficacy of acupuncture in managing chronic pain conditions such as knee osteoarthritis, neck pain, migraines, and lower back pain. Genuine acupuncture has exhibited superior analgesic effects when compared to sham acupuncture and has shown comparable efficacy to widely utilized non-opioid analgesics. These clinical studies were published in renowned medical journals, such as the Journal of the American Medical Association and the British Medical Journal [31–34].

EA provides electrical stimulation to acupoints by setting the parameters of the electrical pulse, which has a broader application prospect than manual acupuncture. However, there remains considerable scope for further exploration into its precise mechanism and underlying materials. The current level of basic research on EA analgesia lags behind clinical research, impeding its modernization and international recognition. There is an urgent need for more comprehensive investigations to bridge this gap.

### AMPK plays a pivotal role in the mechanism of EA-induced analgesia

Numerous studies have demonstrated the multi-target effect of EA in mediating analgesic effects through endogenous analgesic substances, such as endogenous opioids and endocannabinoids. In the 1970s and 1980s, Dr. Han Jisheng’s research found that acupuncture can stimulate the release of endogenous opioids in various regions including the hypothalamus, periaqueductal gray and spinal cord, which can inhibit pain signal transmission [35]. Building on this foundation, our research team in 2009 initially highlighted the critical role of endocannabinoids in EA analgesia, leading to a series of subsequent studies [15–17, 36]. However, the intrinsic connection between endogenous analgesic substances such as opioids and cannabinoids remains unclear at present.

It is encouraging that, through further investigation, AMPK as a new analgesic target may provide new ideas for the elaboration of the intrinsic connection between them. Research has shown that AMPK agonists have analgesic effects in various pain models [8]. Experimental studies have shown that the activation of AMPK can effectively alleviate acute, inflammatory, and neuropathic pain responses by regulating mTOR, which is closely associated with peripheral and spinal hypersensitivity [9, 10, 23]. The results of animal experimental studies have demonstrated that pharmacological activation of AMPK exerts a beneficial or therapeutic effect on inflammatory pain, neuropathic pain resulting from nerve damage, postoperative pain, as well as neuropathic pain induced by chemotherapy or diabetes [8–10]. Preclinical trials indicate that metformin, an AMPK activator, is a widely utilized and well-tolerated pharmaceutical agent, particularly suitable for inclusion in clinical retrospective or prospective studies on pain [8]. These findings strongly suggested that targeting AMPK could be a promising therapeutic approach for managing various types of pain in clinical settings.

Previous studies have demonstrated a strong correlation between EA-induced analgesia and AMPK activation. Acupuncture has been shown to induce hypothalamic AMPK activation in normal rats, leading to an elevation in their pain threshold [37–39]. Besides, the short- and long-term regulatory capacity of AMPK corresponds to the immediate effect and cumulative effect of EA. Based on this, we hypothesize that AMPK could be the pivotal molecule we seek, serving a critical role as both an energy homeostasis sensor and a pain modulator in EA-induced analgesia. Our research has shown that EA robustly activates AMPK in inflamed skin tissue, with no observable activation in the healthy skin tissue on the contralateral side. We observed that EA not only promotes AMPK phosphorylation but also concurrently reduces the inflammatory response in the skin, which may account for its efficacy in alleviating inflammatory pain. The use of an AMPK inhibitor effectively mitigated EA analgesia and the activation of peripheral tissues, specifically within the inflamed skin. This finding further substantiates the essential role of AMPK in mediating the analgesic effects of EA.

### EA stimulates AMPK activation, enhances the expression of β-END in inflamed skin tissue, and attenuates inflammatory pain

Previous studies have shown that AMPK can regulate the polarization of macrophages, promote their shift towards the M2 phenotype, and increase the expression of β-END, which helps to alleviate inflammatory pain [24]. This mechanism is likely due to AMPK's localization in the nuclear chromatin, which enables the transcription of the proopiomelanocortin gene via a p53-dependent pathway, culminating in the production of β-END [40]. It has been hypothesized that EA could potentially regulate the activity of AMPK within macrophages. This modulation may facilitate a shift in the macrophage polarization from a pro-inflammatory M1 state to an anti-inflammatory M2 state. Consequently, this shift could enhance the release of endorphins, which are known for their pain-relieving properties, thereby exerting an overall anti-inflammatory and analgesic effect.

Literature suggests that metformin, an AMPK activator, may alleviate mechanical pain in a paclitaxel-induced neuropathic pain model and the non-selective antagonist of opioid receptors naloxone can attenuate the analgesic activity of metformin. The mechanism is related to the activation of opioid pathways and reduction in TNF-α, IL-1β, and CXCL-1 production in DRG as well as IL-6 production in the thalamus [41]. Another literature review has shown that μ and δ opioid receptors are involved in the analgesic mechanism of EA for neuropathic pain [42]. Therefore, we hypothesize that EA stimulation of AMPK may alleviate pain through the release of β-END and modulation of μ- and δ-opioid receptors, consequently attenuating the production of inflammatory cytokines.

Our study shows that EA enhances AMPK activation in macrophages within inflamed skin tissue, thereby promoting β-END expression. Conversely, inhibition of AMPK can reverse the promotive effect of EA on β-END expression in inflamed skin tissue. These findings further suggested that EA activates AMPK in macrophages within inflamed skin tissue to facilitate β-END expression, consequently reducing the transmission of nociceptive information to the spinal dorsal horn. This downstream mechanism involving peripheral AMPK activation may explain the analgesic effects of EA.

### EA promotes the release of endocannabinoids, activating CB2 receptors on immune cells such as macrophages in inflamed skin tissue, subsequently activating AMPK to exert analgesic effects

The endocannabinoid system plays a pivotal role in skin inflammation and the immune response [43, 44]. It primarily consists of two types of cannabinoid receptors distributed throughout mammalian bodies, namely CB1R and CB2R. Among them, CB1R is predominantly localized in the nervous system, while CB2R is mainly expressed in the immune system, including macrophages, mast cells, T cells, B cells etc. [45].The research presented in this paper demonstrates that EA has the ability to enhance the levels of 2-AG and AEA in inflamed skin tissue, which is consistent with our previous findings [15]. The underlying mechanism behind this phenomenon may involve the depolarization of cell membranes by neural impulses generated by EA, thereby facilitating the hydrolysis of phospholipid precursors catalyzed by phospholipase D and subsequently leading to endogenous cannabinoid production [15]. However, when CB2R was knocked out, EA no longer exhibited an increase in p-AMPK expression in skin tissues. This suggests that EA might activate CB2R in skin tissues via 2-AG and AEA to promote AMPK activation specifically within inflamed skin tissues.

AMPK is a trimeric complex consisting of catalytic subunit α and regulatory subunits β and γ [46]. The primary phosphorylation site for AMPK activation is Thr172 on its α subunit [47]. AMPK plays an important role in the regulation of inflammation, and our work has demonstrated the upstream regulatory effect of CB2R on AMPK in EA analgesia, but the specific intermediate mechanism is not clear. Research has identified that β-caryophyllene induces Ca^2+^/CaMKKβ signaling pathway-mediated activation of AMPK through activated CB2R, which may serve as a downstream signaling mechanism for activating AMPK via CB2R [23]. Several studies have also indicated that the activation of CB2R may stimulate AMPK through alterations in cAMP levels. Christine Börner and colleagues observed that JWH015 and 9-tetrahydrocannabinol activate CB2R, resulting in a transient decrease in cAMP concentration followed by a significant (up to tenfold) and sustained increase for at least 48 h. The modulation of cAMP signaling has the potential to facilitate AMPK activation. Numerous reports have highlighted the involvement of the cAMP/AMPK axis in diverse pathological conditions, including inflammation and ischemia. A recent report has substantiated the role of distinct cAMP microdomains generated by soluble adenylate cyclase in governing AMPK activity [48]. Hence, CB2R may promote AMPK activation by increasing cAMP levels. Therefore, despite variations in signaling pathways and the specific substances that activate CB2R (such as trans-caryophyllene and JWH015), the activation of CB2R ultimately leads to signal transduction by enhancing AMPK activity [20–22].

Research findings have demonstrated the pivotal involvement of hypothalamic AMPK in mediating the analgesic effects of EA, and attenuation of hypothalamic AMPK activation counteracts the pain threshold-enhancing effect induced by EA in normal rats [10]. However, it is noteworthy that the analgesic mechanism of EA for pathological pain differs from that of physiological pain [42]. Currently, there is a lack of clarity regarding the specific cell types in skin tissue activated by AMPK during EA treatment for inflammatory pain. Our study demonstrated that EA effectively activates AMPK in macrophages within inflamed skin tissues. Moreover, when CB2R is knocked out, EA fails to induce an upregulation of phosphorylated AMPK levels in macrophages within inflamed cutaneous tissue, suggesting that EA activates AMPK in macrophages within the inflamed skin tissue by modulating CB2R signaling. This strongly implies that CB2R may serve as an upstream mechanism for EA to activate AMPK and exert its analgesic effect.

Our prior research established a link between CB2 receptors and β-END, revealing that the CB2R agonist AM1241 or EA alleviated inflammatory pain, an effect inhibited by the μ-opioid receptor antagonist β-funaltrexamine. Furthermore, AM1241 or EA enhanced β-END expression in inflamed skin, a response that was counteracted by the CB2R antagonist AM630 [17]. However, our study has limitations, and future investigations should examine whether CB2 gene deletion can suppress the EA-induced upregulation of β-END expression, to further verify the CB2R-AMPK-β-END pathway. Moreover, our previous study found that AMPK activator AICAR reduced inflammatory pain through inhibiting NF-κB activation and IL-1β expression in mice’s inflamed skin [25]. The activation of AMPK by EA may also directly inhibit the expression of IL-1β and exert analgesic effect through CB2R-AMPK-IL-1β pathway as well.

## Conclusion

**Fig. 10:**
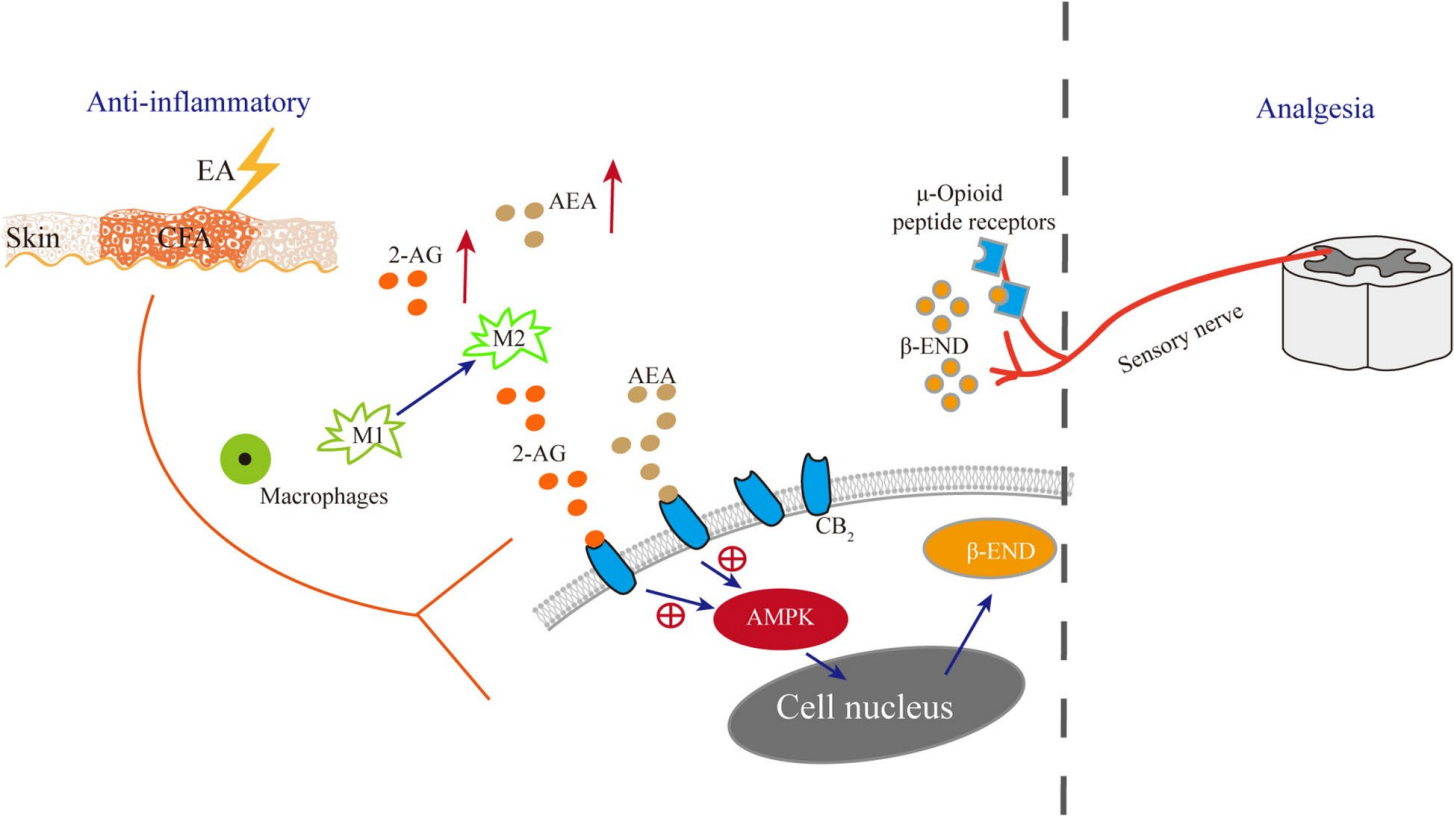
Mechanistic illustration of EA-Induced analgesia via CB2 Receptor-mediated AMPK activation in macropahges of inflamed skin tissue

The current research introduces novel insights into the analgesic mechanisms of EA, demonstrating that it can activate AMPK through the CB2R. This activation enhances the expression of β-END in inflamed skin tissue, thereby desensitizing peripheral sensory nerves via μ-opioid receptors and exerting an analgesic effect (Fig. 10).

Our study presents a novel integration of endogenous cannabinoids and endogenous opioids as two distinct classes of analgesic agents by AMPK, elucidating the endocannbinoid-AMPK-opioid pathway that underlies the analgesic effects of EA.These findings not only enrich our comprehension of EA's therapeutic effects but also hold promise for advancing its clinical application in managing inflammatory pain conditions.

## Supplementary Information

Additional file 1: **Fig. S1.** Electrophoresis of PCR identification of CB2R KO mice; **Fig. S2.** Down-regulated expression of CB2R in dorsal hindpaw skin tissue of CB2R KO mice; **Fig. S3.** Effect of EA on histological features in inflamed skin tissue.

## Data Availability

Not applicable.

## Abbreviations

NSAIDs: Non-steroidal anti-inflammatory drugs
EA: Electroacupuncture
AMPK: Adenosine monophosphate-activated protein kinase
CFA: Complete Freund’s Adjuvant
β-END: β-Endorphin
CB2R KO: CB2 receptor knockout
WT: Wild type
WB: Western blot
PBS: Phosphate-buffer solution
PCR: Polymerase chain reaction
UPLC-MS/MS: Ultra-high performance liquid chromatography tandem mass spectrometry
2-AG: 2-Arachidonoylglycerol
AEA: Anandamide
