# Supplementary material for: Electroacupuncture ameliorates inflammatory pain through CB2 receptor-dependent activation of the AMPK signaling pathway
Source: Chin Med. 2024 Dec 24;19:176. doi: 10.1186/s13020-024-01048-z (PMC11667860; doi:10.1186/s13020-024-01048-z)
Supplement: Supplementary file 1 — Additional file 1: Fig. S1. Electrophoresis of PCR identification of CB2R KO mice; Fig. S2. Down-regulated expression of CB2R in dorsal hindpaw skin tissue of CB2R KO mice; Fig. S3. Effect of EA on histological features in inflamed skin tissue. [file 13020_2024_1048_MOESM1_ESM.docx]

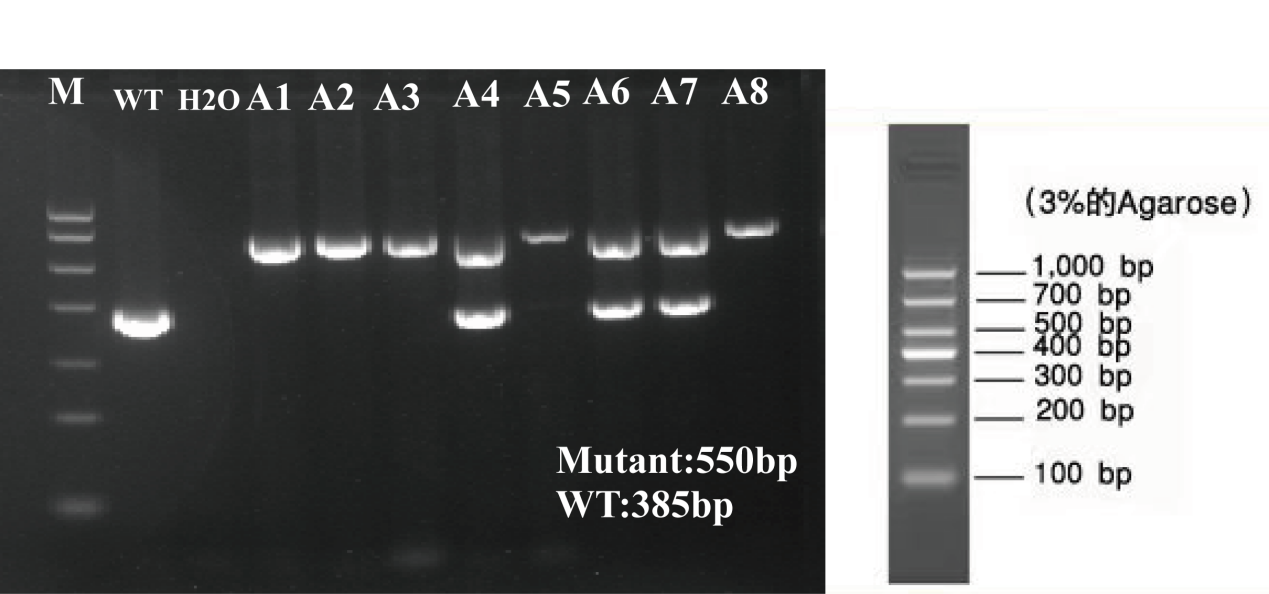


**Supplementary Fig. 1.** Electrophoresis of PCR identification of CB2R KO mice. WT was the wild-type mouse genome, H2O was the negative control, and M was the DNA Marker (TaKaRa, DL1000). Wild-type mouse tail DNA was 385bp, and CB2R KO mouse tail DNA was 550bp. A1-3, A5, and A8 are homozygous mice (CB2R-KO), while A4, A6, and A7 are heterozygous mice.


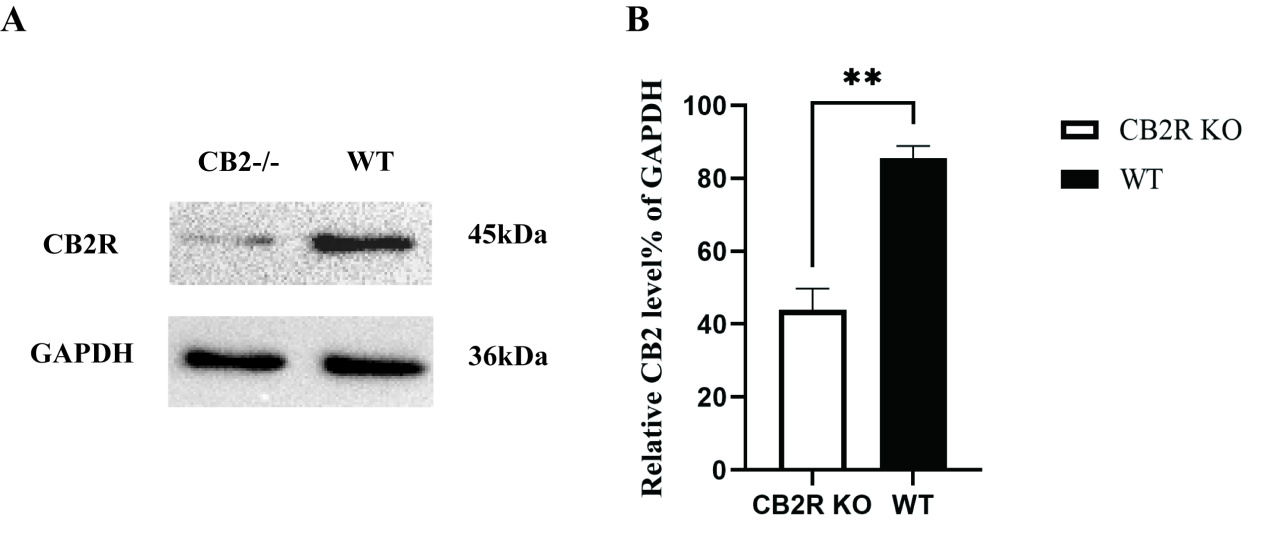


**Supplementary Fig.2.** Down-regulated expression of CB2R in dorsal hindpaw skin tissue of CB2R KO mice. The dorsal hindpaw skin of wild type and CB2R KO mice was tested by WB. **A** Gel representation of CB2R expression levels in the dorsal hindpaw skin of wild-type and CB2R KO mice. **B** Quantitative statistical histogram of the percentage of CB2R to GAPDH. CB2R KO refers to mice with systemic CB2R knockout, and WT refers to wild-type mice. The data is shown as mean ± SEM (n=3). T-test was used to analyze the data. **P < 0.01 compared with WT group.


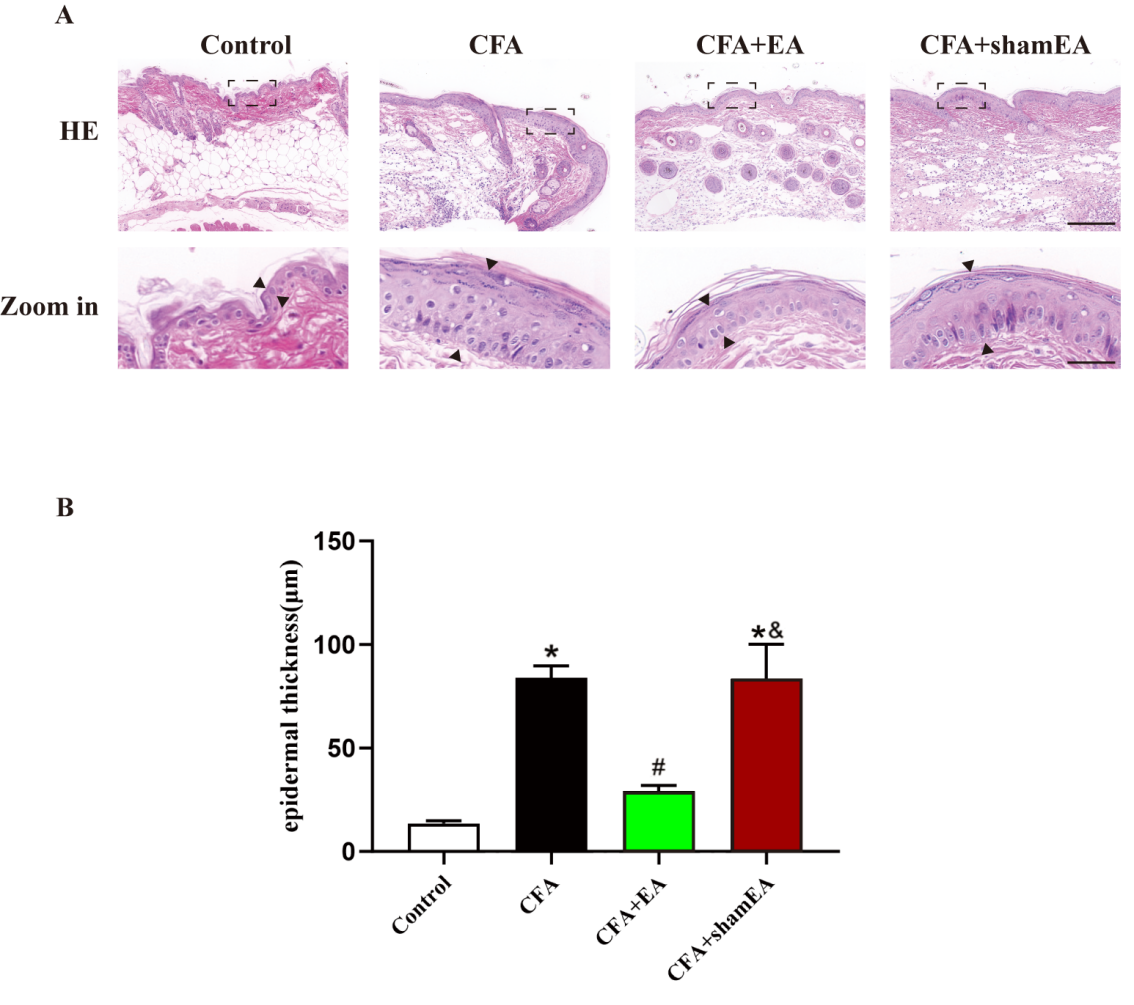


**Supplementary Fig.3.** Effect of EA on histological features in inflamed skin tissue. **A** Representative H&E staining images of each group.The scale bars are 200 μm and 40 μm (Zoom in).**B** Quantitative measurement of epidermal thickness based on the H&E images. The data is shown as mean ± SEM (n=4). One-way ANOVA was used to analyze the data. *P<0.05 compared with Control group; #P<0.05 compared with CFA group; & P<0.05 compared with CFA + EA group.
